# Lentivirus-mediated RNAi silencing targeting ABCC2 increasing the sensitivity of a human nasopharyngeal carcinoma cell line against cisplatin

**DOI:** 10.1186/1479-5876-6-55

**Published:** 2008-10-04

**Authors:** Si Ming Xie, Wei Yi Fang, Zhen Liu, Shuang Xi Wang, Xin Li, Teng Fei Liu, Wei Bing Xie, Kai Tai Yao

**Affiliations:** 1 Cancer Research Institute, Key Lab for Transcriptomics and Proteomics of Human Fatal Diseases Supported by Ministry of Education and Guangdong Province, Southern Medical University, Guangzhou City, Guangdong Province, 510515, PR China; 2 Division of Endocrinology and Diabetes, Department of Medicine, University of Oklahoma Health Sciences Center, Oklahoma City, OK 73104, USA

## Abstract

**Background:**

High resistance to drug is taken as a characteristic of human tumors, which is usually mediated by multidrug resistance-associated genes. ABCC2, an ATP-binding cassette multidrug resistance transporter, is found to be expressed in a variety of human cancers. In this study the effect of a RNAi construct targeting ABCC2 on the chemosensitivity of NPC cell line CNE2 against cisplatin was investigated.

**Methods:**

Lentiviral vectors were constructed to allow an efficient expression of anti-ABCC2 siRNA. The effective target sequence comprised nucleotides 1707–1727 of the human ABCC2 mRNA. The cell clones expressing the construct were picked and expanded, followed by identification using qRT-PCR and western blot method. As control, lentiviral vector containing invalid RNAi sequence was transfected to CNE2 cells. *In vitro*, cellular accumulation of cisplatin was detected by HPLC. The capacity of cellular growth and sensitivity of cells against cisplatin were detected by MTT assay. *In vivo*, the sensitivity of the tumor tissues against cisplatin were evaluated by transplanted CNE2 nude mice model.

**Results:**

Two CNE2 cell clones with reduced expression of targeted ABCC2 mRNA and protein for more than 70% by qRT-PCR and western blot were established, and no differences were shown in proliferation rates compared to control CNE2 cells by growth curves analysis. *In vitro *the accumulation of intracellular cisplatin in these CNE2 cell clones with reduced expression of ABCC2 increased markedly, accompanied by increased sensitivity against cisplatin. *In vivo*, the growth of CNE2 solid tumors with a stably transfected anti-ABCC2 siRNA construct was significantly inhibited by cisplatin in transplanted nude mice model.

**Conclusion:**

Our investigation demonstrated that lentivirus-mediated RNAi silencing targeting ABCC2 might reverse the ABCC2-related drug resistance of NPC cell line CNE2 against cisplatin.

## Background

Nasopharyngeal carcinoma (NPC) is a common malignant epithelial tumor in Southern China with an unusually high incidence (10–150/100,1000 per year)[1]. NPC originates from a hidden anatomical site, and is more closely associated with advanced clinical stage with higher incidence of invasion and metastasis at the time of presentation to the first biopsy. Therefore, chemotherapy treatment is a necessary ancillary method for these NPC patients [2-4]. Of all the chemotherapy drugs, cisplatin is the most effective cytotoxic agent used in NPC treatments. However, inherent and acquired resistance to the drug limits its applications in NPC chemotherapy, which may account for the failure of chemotherapy for patients with advanced NPC. Currently much interest in the mechanisms responsible for cisplatin-resistance is given, but none is fully understood. Reduction in cellular accumulation of cisplatin is one of the principal mechanisms of resistance, which may be ascribed to an increase in drug efflux. The adenosine triphosphate binding cassette (ABC) transporter families, whose products represent membrane proteins, have the capability to use energy to drive the transporters of various molecules across the cellular membrane, and are confirmed to be associated with anticancer drug transporter [5,6].

Of all the ABC transporters, ABCC2, also designated MRP2 or cMOAT, had been identified to confer cellular resistance of tumor cells to various anticancer drugs including cisplatin [7]. A 10-fold increase in resistance has been demonstrated in cells overexpressing MRP2 by gene transfection [8]. The increased level of ABCC2 mRNA in some human carcinoma cell lines was associated with relative cisplatin resistance owing to reducing intracellular accumulation of cisplatin and decreasing DNA adduct formation[7,9-11]. On the other hand, reduced expression of ABCC2 mRNA could increase the sensitivity of these cells against cisplatin [12-14]. Interestingly, Pawel [8] found that ABCC2 can be localized in the nuclear membrane of ovarian carcinomas, which was associated with response to chemotherapy. Given that DNA is the primary target of cisplatin [5], this finding strongly indicates that there is a close relationship between ABCC2 expression and cisplatin-resistance.

Until now, there was never any evidence that has shown a relationship between ABCC2 expression and cisplatin-resistance in NPC. In this investigation, small interfering RNA (siRNA) technique using lentivirus vector was applied to specifically inhibit the expression of ABCC2 in a NPC cell line CNE2, and HPLC was used to detect the intracellular accumulation of cisplatin, followed by determination of cisplatin cytotoxicity. Finally *in vivo *model was used to evaluate the efficacy of cisplatin to transplanted tumors.

## Methods

### Cell lines and animals

The human NPC cell lines CNE1, CNE2, 5–8F, 6–10B, and HONE1 were grown in RPMI-1640 medium (Hyclone, Logan, UT) supplemented with 10% fetal calf serum (ExCell, Shanghai, China) and 1% L-glutamine [15]. NP69, a human immortalized nasopharyngeal epithelial cell line, was grown in defined-KSFM medium supplemented with EGF (Invitrogen, Carlsbad, CA) [16]. Human embryonic kidney cell line 293FT was grown in DMEM supplemented with 10% fetal calf serum (Hyclone, Logan, UT) [17]. All cell lines were cultured at 37°C in a humidified atmosphere of 5% CO_2_. BALB/c nude mice, 4–6-weeks-old, weighing 18–22 g at the start of the study, were used.

### Detection of ABCC2 mRNA levels in NPC cells by Quantitative RT-PCR

Expression of ABCC2 mRNA in NPC cell lines was detected compared to that in NP69 cell line. Total RNA was isolated by using Trizol reagent (Invitrogen, Carlsbad, CA) according to the manufacturer's instructions. Quantitative RT-PCR was carried out using a MX3000P instrument (Stratagene, Cedar Creek, TX) and SYBR^® ^Premix Ex Taq™ kit (Takara bio, Otsu, Japan) to detect the mRNA level of ABCC2, with ACTB (β-actin) as a normalizing control. The specific PCR primer sequences of these genes designed by Primer premier 5.0 software were as follow: ABCC2 forward: 5'-CTC ACTTCAGCGAGACCG-3'; ABCC2 reverse: 5'-CCAGCCAGTTCAGGGTTT-3'; ACTB forward: 5'-CACCCAGCACAATGAAGAT-3'; ACTB reverse: 5'-CA AATAAAGCCATGCCAAT-3'. Cycling conditions were used as described previously [17]: 95°C for 10 min to activate DNA polymerase, followed by 45 cycles of 95°C for 15 s, 55°C for 20 s, and 72°C for 10 s. Specificity of amplification products was confirmed by melting curve analysis. Independent experiments were done in triplicate. For mRNA quantification, samples were normalized against the expression of ACTB mRNA. The NPC cell line with the highest expression of ABCC2 was chosen for the next step.

### Design of anti-ABCC2 RNAi sequence and construction of shRNA expressing vectors

Two different ABCC2-specific target sequences were chosen according to online siRNA tools of Invitrogen  using the ABCC2 reference sequence (Gene Bank Accession No. NM_000392.2). The target sequences of ABCC2-A (5'-GCTGGCCTTTAGTCAACTACA-3') and ABCC2-B (5'-GCAGCTGGATTACATGCTTCC-3') are homologous to nt 1707–1727 and 3398–3418 of the ABCC2-specific mRNA, respectively, followed by shRNAs chemically synthesized and lentivirus vector constructed as described previously [18], with invalid RNAi sequence (5'-GCAGGAGCTATGCTACCATCA-3') as negative control. The correct insertion of the specific shRNA was further confirmed by sequencing.

### Treatment of CNE2 cells with shRNA-encoding expression construct

The ABCC2-specific shRNA-encoding expression construct and optimized ViraPower™ Packaging Mix were co-transfected to 293FT cell line using the lipofectamine 2000 (Invitrogen, Carlsbad, CA) to produce lentivirus stock, with ABCC2-shRNA negative construct as negative control. After the titer was determined, the lentivirus stock was transfected to NPC cell line CNE2 according to the manufacture recommendations of BLOCK-iT™ Lentiviral RNAi Expression System (Invitrogen, Carlsbad, CA). For stable silencing of ABCC2, the transfected CNE2 cell line was selected by blasticidin, followed by blasticidin-resistant colonies picked, expanded and analyzed separately. By using MTT (3-(4, 5-dimethylthiazol-2-yl)-2, 5-diphenyltetrazoliumbromide) (Sigma, Louis, MO, USA.) cell viability assay, routine checks of the cell growth for 7 days were performed to assess the viability of the transfected cells [18].

### Analysis of ABCC2 mRNA levels by quantitative RT-PCR

Total RNA of these cell clones was isolated and quantitative RT-PCR was performed to detect the mRNA level of ABCC2 as described above. Each sample was measured at least three times.

### Analysis of ABCC2 protein levels by western blot

After proteins of all cells were prepared, western blot method was performed as described previously [18]. Rabbit anti-ABCC2, with mouse anti-beta-actin (Boster, Wuhan, China) as normalized control, was used to detect the modulation of ABCC2 protein level, accompanied by the analyses of the *Image-Pro^® ^Plus *software.

### Drug accumulation assays by high performance liquid chromatograph

One day after cells were seeded in 25 cm^2 ^flasks, cisplatin (10 μg/ml) was added to the flasks. Two hours later, cells were harvested and counted, with total 10^6 ^cells to be used. After washing by RPMI-1640 for three times, cell pellets were collected and resuspended in 0.3 ml distilled water, followed by freeze/thaw for 5 times to breakdown the cells. After centrifuging at 12,000 r.p.m for 30 minutes, supernatant was collected to be used. Detection of cisplatin was performed by high performance liquid chromatograph (HPLC) (series 1200, Agilent, Santa. Clara, CA, USA) as described previously [19]. Cisplatin standard solutions ranging from 5–80 μg/ml were used for preparation of calibration curve.

### Cytotoxicity assays by MTT

Sensitivity of cells against cisplatin was determined using the MTT assay. Cells were seeded in 96-well plates at a density of 5 × 10^3 ^cells per well. Various gradient concentration of cisplatin (Qi Lu Pharmaceutical Factory, Jinan, China) ranging from 0.5–32 μg/ml were added to each well 24 hours after seeding. After 48 hours of incubation under normal culture conditions, MTT was added at a final concentration of 5 mg/ml. Four hours later, DMSO (Sigma, Louis, MO, USA.) was added to dissolve the crystal with shaking horizontally for ten minutes [20]. The OD value of 570 nm wavelength was measured by microplate reader (Bio Rad, Hercules, CA). The IC50 value, defined as the drug concentration required to reduce cell survival to 50% determined by the relative absorbance of MTT, was assessed by probit regression analysis in SPSS11.5 statistical software.

### *In Vivo *Treatments

Cells were cultured in 75 cm^2 ^flask. Cell suspensions of 10^6 ^cells were transplanted subcutaneously in BALB/c nude mice. A tumor mass of 50 mg was evident in all mice on day 7 after transplantation. The mice were divided into two groups, with 5–8 animals in each group. The animals in group 1, as normalized control, received physiological saline i.p., and group 2 received cisplatin i.p. at 3 mg/kg once a week for 3 continuous weeks. The tumor weight was evaluated at day 4, 7, 11, 14, 18 and 21. Two orthogonal diameters of the tumors were measured with vernier calipers. The following formula for measuring the tumor weight was used: *Weight *= (*length *× *width*^2^*)/2 *[21]. Tumors with weights outside the range of 50–200 mg at the start of the treatment were excluded from the study. After exclusion, there were 4–6 animals left in each group. On day 21 after treatment, all mice were killed and the tumor tissues were collected and fixed in 10% formalin. Immunohistochemistry method was used to detect the expression of ABCC2 in tumor tissues. Relative tumor size (RTS) was calculated as the tumor volume at the time of measurement divided by that of treatment. The mean RTS value was plotted as function of time for various treatment groups. Tumor growth inhibition was determined as the ratio of treated: control (T: C), which was calculated as mean RTS of cisplatin treatment groups divided by the mean RTS of normal saline groups. The T: C value of <42% is the minimum level for determining that a treatment regime has activity [21].

### Statistic analysis

The data of quantitative RT-PCR, MTT, IC50, intracellular accumulation of cisplatin and the relative tumor size were expressed as mean ± SD value. Statistical analysis for relative tumor size were carried out by repeated measures, and other data by ANOVA, with LSD test for multiple comparisons in statistical package SPSS 11.5. The results were considered statistically significant at *P *< 0.05.

## Results and discussion

### ABCC2 mRNA is highly expressed in CNE2 cell line

**Figure 1:**
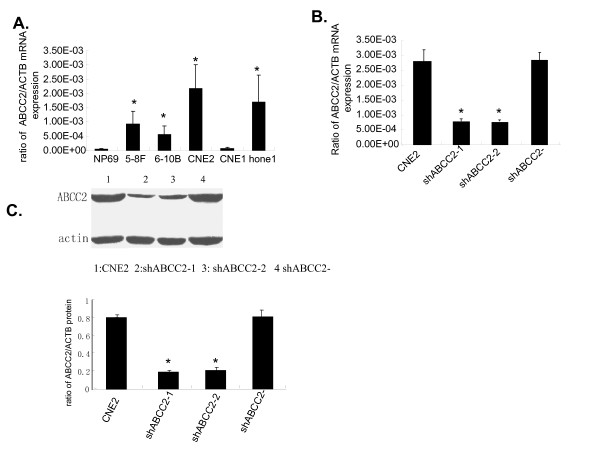
**Special siRNA targeting ABCC2 silences the mRNA and protein expressions of ABCC2 in NPC cells**. (A) Expression of ABCC2 mRNA in NP69 and human NPC cell lines by quantitative RT-PCR. N = 3, *P < 0.05 *vs*. NP69. (B and C)Analysis of ABCC2 expression levels in CNE2 cells treated with ABCC2-shRNA construct (ShABCC2-1 and shABCC2-2) and negative construct (ShABCC2-). (B) The expressions of ABCC2 mRNA detected by quantitative RT-PCR. (C) The expressions of ABCC2 protein detected by western blot. Data were expressed as mean ± SEM value. N = 3, *P < 0.05 *vs*. CNE2.

ABCC2 is normally expressed on the apical membrane of hepatocytes, and encodes a major organic anion transporter in the canalicular membrane of hepatocytes [11]. In human cancer cell lines including head and neck squamous cell carcinoma, ovarian carcinoma, hepatoma, and so on, the expression of ABCC2 has also been found [11,12]. However, little research on the expression of ABCC2 in NPC cell lines has been reported. In this investigation, the expression of ABCC2 mRNA was found in NPC cell lines, with the highest expression in CNE2 cell line compared to human immortalized nasopharyngeal epithelial cell line NP69 by quantitative RT-PCR method (Fig. 1A), which indicated that CNE2 cell line is a suitable cell model for RNAi targeting ABCC2 mRNA.

### Lentivirus-mediated RNAi silencing inhibited the expression of ABCC2 mRNA and protein in CNE2 cell line

It has been demonstrated that RNAi can achieve effective, stable gene silencing in diverse biological systems and will assist in elucidating gene functions in numerous cell types including primary cells [22]. Two different siRNA constructs, ABCC2-A and ABCC2-B, were used to silence the mRNA expression of ABCC2 in CNE2 cell line, with negative construct as control (named as CNE2/shABCC2-). As a result, the gene silencing efficacy of ABCC2-A was stronger than that of ABCC2-B (data not shown). After ABCC2-A siRNA construct was transfected into CNE2 cells, twelve cell clones with stably expressed ABCC2-shRNA were picked, cultured and analyzed separately. ABCC2 mRNA expression in these cell clones was compared to that in parent CNE2 cells and negative control (CNE2/shABCC2-) by quantitative RT-PCR. As a result, two cell clones showed decreased level of ABCC2 mRNA expression for about 72%, and named as CNE2/shABCC2-1 and -2, respectively (Fig. 1B).

To further confirm the specificity of siRNA-mediated silencing of ABCC2, the detection of ABCC2 protein expression of the selected cell clones was determined by western blot. As shown in Fig. 1C and 1D, ABCC2 protein expression of both cell clones, CNE2/shABCC2-1 and -2, was decreased by 76% and 74%, respectively.

The results of quantitative RT-PCR and Western blot assays revealed that expression of ABCC2 in two selected cell clones was markedly decreased, which demonstrated that RNAi technique was an effective way to modulate the ABCC2 expression in CNE2 cell line. The selected cell clones, CNE2/shABCC2-1 and -2, were used as the knock-down cell model of ABCC2 in subsequent experiments.

The cellular target of cisplatin has long been believed to be DNA, for it has been shown to bind DNA and cause the DNA duplex to bend and unwind. Interestingly, it had been reported that after treatment with RNAi targeting ABCC2, decreased nuclear membranous ABCC2 protein expression in the cisplatin-resistant cancer cell lines was also observed [14]. As demonstrated previously, ABCC2 is localized in the nuclear membrane of cisplatin-resistant cells, and nuclear membranous localization of ABCC2 correlated with resistance against cisplatin in ovarian carcinoma cells [11,23]. Thus, ABCC2 may protect the nucleus from formation of platinum-DNA adducts by driving cisplatin out of the nucleus. However, it still need to be studied in CNE2/shABCC2 cell clones.

### Down-regulated expression of ABCC2 by siRNA increased the intracellular accumulation of cisplatin

**Figure 2:**
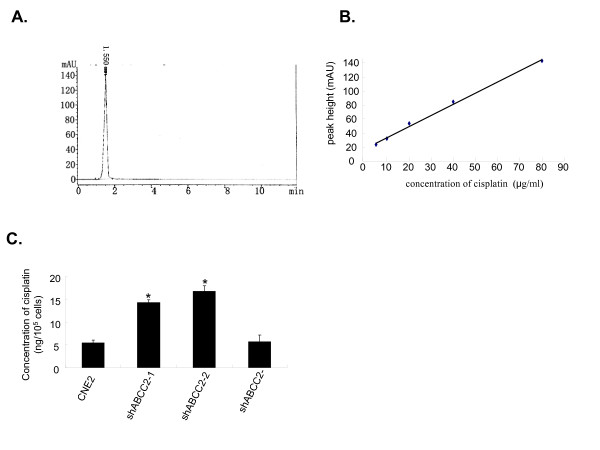
**ABCC2 siRNA increased the intracellular accumulation of cisplatin**. (A) A typical chromatogram for total analysis of cisplatin from CNE2 cells exposed to cisplatin for 2 h using HPLC determination. (B) Calibration curve for gradient concentration of cisplatin within the range of 5–80 μg/ml. A typical linear relationship (R^2 ^= 0.9965) was found between peak height and concentration of cisplatin. (C) The cellular accumulation of cisplatin in CNE2 cells treated with ABCC2-shRNA construct (ShABCC2-1 and shABCC2-2) and negative construct (ShABCC2-). The concentration of cisplatin were determined according to the calibration curve of cisplatin. Data were expressed as mean ± SEM value. N = 3, *P < 0.05 *vs*. CNE2.

Atomic absorption spectroscopy has been the most commonly used technique for cisplatin determination. However, this procedure involves complicated handling of the samples. High performance liquid chromatography (HPLC) is a rapid, economic and validated way to determine the accumulation of cisplatin in plasma, cancer cell and tumor samples [24]. Therefore, HPLC was used to detect the cellular accumulation of cisplatin in CNE2 cells. As shown in Fig. 2A, a symmetrical peak for typical chromatograms of cisplatin was shown, and retention time for the cisplatin was about 1.55 min. A typical linear relationship (R^2 ^= 0.9965) was found between peak height and gradient concentration of cisplatin (Fig. 2B). The equation obtained from this calibration curve was *y *= 1.59*x *+ 17.917(*y *stands for peak height of cisplatin and *x *stands for concentration of cisplatin). Based on this equation, the concentration of cisplatin for each sample was determined. As a result, intracellular accumulation of cisplatin in CNE2/shABCC2-1 and -2 cell clones were enhanced by 2.66 and 3.11 folds respectively (Fig. 2C). These data showed that intracellular accumulation of cisplatin in CNE2 cells with decreased expression of ABCC2 was more than that in parent CNE2 cells, which indicated that ABCC2 protein has the capacity to drive cisplatin out of the CNE2 cells.

### ABCC2 siRNA increased the sensitivity of cisplatin in CNE2 cells without changing the cell viability

**Figure 3:**
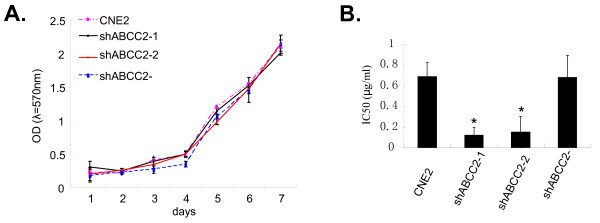
**Silencing of ABCC2 by siRNA increased the sensitivity of cisplatin in CNE2 without changing the cellular viability**. (A) Cellular growth curve. The cell growth viability was assessed by MTT method for 7 days. (B) Modulation of sensitivity against cisplatin for CNE2 cells. MTT method was used to determined the IC50 value of cisplatin to CNE2 cells treated with ABCC2-shRNA construct (ShABCC2-1 and shABCC2-2) and ABCC2-shRNA negative construct (ShABCC2-). Data were expressed as mean ± SEM value. N = 3, * *P *< 0.05 *vs*. CNE2.

To assess the cell viability, CNE2, CNE2/shABCC2-1, CNE2/shABCC2-2 and CNE2/shABCC2 cells were seeded onto 96-well microplates. Cellular growth was determined by a continuous 7-day MTT assay, and growth curve was made according to these OD values alterations of MTT assay. No significant difference was found between the cell growth of these cells (Fig. 3A), which indicate that the viability of cells was influenced neither by the transfection procedure, nor by ABCC2 gene.

To evaluate the sensitivity against cisplatin, these cells were seeded onto 96-well microplates and various concentrations of cisplatin (0.5–32 μg/ml) were added to each well, followed by MTT assay and cell growth inhibition rate determined. Sensitivity of CNE2/shABCC2-1 and -2 against cisplatin was increased by 83% and 78%, respectively, compared to control cells in terms of IC50 (Fig. 3B). Interestingly, a similar result was also shown in the cisplatin-resistant ovarian carcinoma cell line with the treatment of RNAi targeting ABCC2 [14]. Here, our *in vitro *data suggest that reduced ABCC2 expression can influence the cytotoxicity of cisplatin to CNE2 cells by increasing intracellular accumulation of cisplatin.

### *In vivo *antitumor effect of cisplatin

It is well known that many solid tumor are not sensitive to chemotherapy in clinic, which may be ascribed to abnormal expression of multidrug resistance associated genes. ABCC2 is an ATP-binding cassette transporter mediating multidrug resistance of cancer chemotherapy. Although there is a notable correlation between the increased sensitivity of cisplatin and decreased ABCC2 expression in CNE2 cell line *in vitro*, *in vivo *antitumor effect of cisplatin using nude mice model had to be further evaluated.

**Figure 4:**
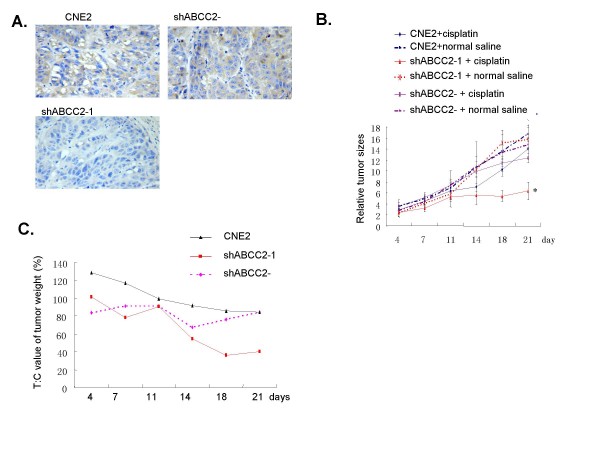
**Silencing of ABCC2 by siRNA increased the inhibitory effects of cisplatin on growth of tumors in nude mice**. (A) Expression of ABCC2 in tumor tissues (Immunohistochemistry method, DAB staining, 200×). CNE2, CNE2/shABCC2-1 cells were transplanted s.c. in nude mice, with ABCC2-shRNA negative construct (ShABCC2-) as control. (B) Efficacy of cisplatin on growth of tumors transplanted s.c. in nude mice. The efficacy was evaluated by relative tumor sizes (RTS). At the time of cisplatin administration, the weight of tumors were in the range of 50–200 mg. Data were expressed as mean ± SEM value. * *P *< 0.05. *P *value were shown in Tab 1. (C) The treated: control (T: C) ratio of tumor weight for s.c. tumors in nude mice. CNE2, CNE2 treated with shABCC2 construct (shABCC2-1) were used, with shABCC2 negative construct (shABCC2-) as control.

CNE2/shABCC2-1 cell line, which was more sensitive against cisplatin, was applied to this model. The expression of ABCC2 in parent CNE2 and CNE2 negative control solid tumors were positive, while in CNE2/shABCC2-1 solid tumor it was weak (Fig. 4A), which indicate that RNAi technique targeting ABCC2 is also effective in nude mice model. The solid tumor of CNE2/shABCC2-1 with weak positive ABCC2 expression was more sensitive to cisplatin treatment than that of parent CNE2 and negative control with positive ABCC2 expression. The growth speed of CNE2/shABCC2-1 tumor after cisplatin treatment was inhibited compared to other two control group (*P *< 0.05) (Fig. 4B and Tab. 1). After cisplatin was administered, the tumor growth was arrested from 18 days on, with T: C value for CNE2/shABCC2-1 smaller than 42% (36% for 18-day and 40% for 21-day) which is the minimum level for determining that a treatment regime has activity (Fig. 4C). These results suggest that ABCC2 protein can efficiently mediate the sensitivity of cisplatin to CNE2 cell line *in vivo*.

**Table 1:** *P *value for comparisons of relative tumor size(RTS) between each group by LSD test.

group	1	2	3	4	5	6
1	-	0.23	0.00056	0.72	0.52	0.77
2	0.23	-	0.048	0.42	0.69	0.47
3	***0.00056***	***0.048***	***-***	***0.0042***	***0.028***	***0.012***
4	0.72	0.42	0.0042	-	0.75	0.99
5	0.52	0.69	0.028	0.75	-	0.77
6	0.78	0.47	0.013	0.99	0.77	-

Note: 1, animals with CNE2 treated with cisplatin. 2, animals with CNE2 treated with normal saline. 3, animals with CNE2/shABCC2-1 treated with cisplatin. 4, animals with CNE2/shABCC2-1 treated with normal saline. 5, animals with CNE2/shABCC2- treated with cisplatin. 6, animals with CNE2/shABCC2- treated with normal saline.

Cisplatin is one of the most used drugs in chemotherapeutic treatment for NPC. Our results including *in vitro *and *in vivo *data indicate that ABCC2 may play an important role in modulating the response of CNE2 cell line against cisplatin. Thus, targeting ABCC2 may be a promising strategy to overcome the cisplatin-resistance in NPC. Because of the effectiveness and specificity of RNAi technology, therapeutic approach of siRNA targeting ABCC2 gene may be applicable in preventing and reversing ABCC2-depending cisplatin resistance in NPC.

## Conclusion

In conclusion, siRNA targeting ABCC2 can markedly reduce the expression of ABCC2 mRNA and protein, which results in an increased intracellular accumulation of cisplatin in NPC cell line CNE2 and noticeably enhances the sensitivity of CNE2 cells against cisplatin. Our *in vivo *model also confirmed that after treatment with cisplatin, the growth speed of tumor of the ABCC2-knockdown CNE2 cells was markedly slower compared to that of parent CNE2 cells and CNE2 cells with negative control construct. These results suggest that ABCC2 may play an important role in NPC resistant to cisplatin.

## Competing interests

The authors declare that they have no competing interests.

## Authors' contributions

All authors have read and approved the final manuscript, SMX set up the protocols, WYF and ZL contributed in the experimental procedures and in the interpretation of the data, SXW, XL, TFL and WBX gave advises on the work and helped in the interpretation of the data, KTY supervised all the work and wrote the paper together with SMX, WYF.
